# The clinical features, diagnosis and management of recurrent thymoma

**DOI:** 10.1186/s13019-016-0533-9

**Published:** 2016-08-31

**Authors:** Taobo Luo, Hongguang Zhao, Xinming Zhou

**Affiliations:** 1 Department of Thoracic Surgery, Zhejiang Cancer Hospital, Hangzhou, 310022 People’s Republic of China; 2 Wenzhou Medical University, Wenzhou, 325035 People’s Republic of China; 3 Zhejiang Key Laboratory of Diagnosis and Treatment Technology on Thoracic Oncology (Lung and Esophagus), Zhejiang Cancer Hospital, Hangzhou, 310022 People’s Republic of China

**Keywords:** Thymoma, Recurrence, Risk factor, Diagnosis, Treatment, Prognosis

## Abstract

Thymoma is a disease with malignant potential, which has a recurrence rate after complete resection ranging from 5 to 50 %. Multiple studies on the risk factors, treatment or prognosis have been reported. Many of them are controversial, however. In this review, we summarized some accepted risk factors, means of diagnosis and different treatments of recurrent thymoma. The risk factors of recurrent thymoma haven’t been well-studied, and its management remains controversial. We reviewed the literatures and found some key points which should be noticed during the surgery of initial thymoma. Although reoperation should be taken into account preferentially, multimodal treatments are also available. The prognosis are also been discussed.

## Background

Thymoma is a disease with malignant potential, which shows a possibility of recurrence after complete resection. The International Thymic Malignancy Interest Group (ITMIG) has recently defined a standard set of definitions for recurrence [1]: (1) the term ‘recurrence’ is appropriate if all disease has been potentially eradicated (R0 resection); (2) recurrences are classified as local (anterior mediastinum), regional (intrathoracic not contiguous with the thymus), and distant (intrapulmonary and extrathoracic); and (3) the freedom-from- recurrence outcome indicator should be used for any study on recurrence after R0 resection, and 5- and 10-year outcomes should be reported in every series.

The recurrence of thymoma is rare, the rate ranges according to different reports. In the research of Japanese Association for Research on Thymus (JART), among all the 2835 thymoma patients received operation during 1991–2010, 420 (14.8 %) experienced recurrence [2]. The average disease-free time of recurrent patients was 5 years, and recurrence occurred 32 years after initial operation was also reported [3]. The time to relapse was 10 years for patient of clinical stage I, and 3 years for patient of stage II, III and IV.

Most recurrence are local and regional [4, 5]. 46–80 % of recurrent cases are found in the thoracic cavity [5–7], and then in the mediastinum and lungs [2, 8], distant metastases occur in less than 5 % of the cases [9]. In the report of Detterbeck et al. [10], Among patients with recurrences, the pleural space or the lung was involved in 58 % (most often as a nodule under the parietal pleura), the pericardium or mediastinum in 41 %, bone in 10 %, and liver in 8 %.

According to the study of The Japanese Association for Chest Surgery [11], of 862 patients who had information, 67 (7.8 %) developed recurrence. The recurrence rates in stages I, II, III, and IV were 0.9, 4.1, 28.4, and 34.3 %, respectively; In thymic carcinoma, 51 % of patients developed recurrence, whereas, in thymic carcinoid, 64 % of patients had recurrence.

## The risk factors of recurrent thymoma

### Masaoka classification

The Masaoka classification was one of the well-accepted risk factors of thymoma recurrent. Tumors capsulated and not infiltrated with neighbor tissue or organs recur very rarely (0 to 5 %) [12]; invasive tumors show a recurrence rate ranging from 20 to 50 % from stage 1 to stage 4a, respectively [11, 13]. Most stage I and II thymomas recur locally or regionally [14]. The majority of the recurrent patients had advanced disease of stage III or IV (*n* = 329; 81.2 %) [2]. Some researchers believe that the invasion of major blood vessel (stage IIIb) strongly suggest tumor recurrence [15]. The more advance Masaoka stage is, the more possible remote recurrence happens.

### World Health Organization histology

The recurrence of thymoma is closely related to its World Health Organization (WHO) histology. It is reported that few patients of type A/AB thymoma experience recurrence, when the recurrent rate was 28.6 % and over 50 % for patients with type B and type C thymoma, respectively [11]. The recurrence of type C thymoma is quicker and more frequent than the other types; its metastasis is also much common [16, 17]. Wright et al. [7] raised a point of view that if divide thymoma among four groups (A/AB, B1/B2, B3 and C) according to WHO histology, its rate of local invasion and recurrence rises one by one. The recurrent rate of type C thymoma is several times higher than the others (41 % vs 9.7 %) [17]. There is some relationship between the WHO histology and Masaoka classification of thymoma, as most type A and type B thymoma are at stage I and stage II, and type B2/B3/C thymoma are usually at stage III and stage IV [8].

### Other risk factors

Incomplete initial resection is regarded as one of the risk factors of recurrence. Taken the possibility of ectopic and vagus thymus into consideration, a synchronous tumor resection and clean-up of the fat tissue in anterior mediastinum is helpful to avoid the recurrence of thymoma, in spite of the tumor’s volume and the integrity of its envelope [18, 19]. The method of initial operation may also be a risk factor. Some researchers raised concern about whether minimally invasive (Video assisted thoracic surgery or Da Vinci robot) methods is prone to induce recurrence [20, 21], but recent reports indicated that there isn’t significant difference between minimally invasive surgery and traditional open operation for experienced surgeons [22, 23]. In case of thymomas greater than 2 cm and without fat tissue surrounding the thymic capsule, the manipulation of the tumor may cause seeding of tumor cells that are responsible for local and pleural recurrences [16]. Recurrence may occur within the thymus left in place during the first surgical procedure or in the thoracic compartment where thymoma was located, in the pleural space opened to allow complete resection, and even at the site of the wound of the first operation or the mediastinal biopsy initially performed for tissue diagnosis [24], which suggests the risk of implantation metastasis during the operation. Other researchers find that tumor larger than 8 cm is also a risk factor of recurrence [7, 25]. Otherwise, studies on the molecular biology discover that DNA aneuploidy, IFN-αand IL-12 are also helpful in predicting the recurrence of thymoma [26–28].

## The diagnosis of recurrent thymoma

### Symptoms

The recurrence of thymoma is usually insidious, most patients have no complains [29]. Myasthenia gravis is most common among all the symptoms, which may appear on more than half of the patients [17, 30]. One patient who shows the symptom of myasthenia gravis induced by the initial thymoma, may be also caught by it when tumor recurrent [31]. In addition, some patients could have oppress symptoms such as chest pain and dyspnea, which are like initial thymoma.

### Computerized tomographic scanning

Computerized tomographic scanning (CT) is the routine examination for patients after initial tumor resection, and is also the main way to screen recurrence thymoma. A research shows that 94 % of asymptomatic patients found the mass by CT [32]. If myasthenia gravis or other symptoms arises, CT could be the first choice. However, CT is a morphological examination; and local tissue adhesion, fibrillation and scarring induced by surgery or post-surgery radiotherapy may harass the interpretation of CT images.

#### Positron emission tomography

Positron emission tomography (PET-CT) could make up the shortness of CT by measuring the metabolic rate of tissue, which improve the accuracy of diagnosis. CT overall sensitivity for detecting the mediastinal recurrence and pleural dissemination of thymomas is 71 %, and its specificity is 85 %; as for PET/CT, the overall sensitivity and specificity for thymoma recurrence are 82 and 95 %, respectively [33]. When employed for the diagnosis of thymoma recurrence in the anterior mediastinum, the sensitivity of PET-CT has reached 100 %, when CT is only 55 % [33]. Besides that, the metabolic rate of tissue showed by PET-CT is related to the grade of malignancy of recurrent thymoma [34]. PET-CT may even help to classify the World Health Organization histology of recurrent thymoma [35]. Nevertheless, the recurrence thymoma on the pleura usually appears as multiple nodules in the image of PET-CT, and the calculation of metabolic rate is difficult because of partial volume effect [36].

#### Others

Magnetic resonance imaging (MRI) is a means with low radiation dose, and is better at representing cystic tissue than CT; MRI also has an advantage in differentiating thymic hyperplasia and thymoma, even evaluating whether the phrenicus nerve is infiltrated [37]. Literature states that ECT shows increased uptake of Tc-99 m in the primary and recurrent thymoma [38], which indicates ECT may be useful in the diagnosis.

### Pathology

Pathology is the gold standard of tumor diagnosis, which consists cytopathology and histopathology. As in most cases, the tumor locates in the anterior mediastinal, sheltered by sternum and surrounded by main vessels, so the puncture guided by CT or ultrasound could be performed only if the tumor spreads out of the sternum. Histopathology could be performed through mediastinoscope, thoracoscope or open surgery.

## The prevent of recurrent thymoma

### In the operation

The principle of tumor-free is crucial for the prevention of local and regional prevention. Complete resection of the entire thymus is recommended, along with extreme care during dissection and en bloc resection of the surrounding structures infiltrated by the tumor [8]. Blockade of the tumor surface could reduce the risk of tumor implantation metastasis.

Most recurrence are found pleural recurrence, it is not clear whether they are related to the particular biology of the tumor or whether they are consequences of seeding of the tumor, possibly determined by disruption of the capsule during the operation. For this reason, some surgeons recommend to avoid opening the mediastinal pleura and avoid miniinvasive procedures with a transpleural approach (thoracotomy, VATS, or robot assisted) [14, 15].

### Postoperative

As quite a bit of recurrent thymoma are in the mediastinum, so there are viewpoints that mediastinal radiation after initial operation are effective in reducing the recurrent rate [6]. However, subsequent reports showed that adjuvant radiotherapy was associated with late morbidity to the heart, lungs, and other mediastinal structures [39–41]; the cost of radiation therapy is sometimes higher than surgery, and mediastinal radiation does not prevent pleural implants, which represents the most common pattern of recurrence [8], so selective use of radiation therapy was suggested.

Currently, adjuvant radiotherapy is not indicated for stage I thymoma, as it’s easy to achieve R0 resection, and histology is usually type A or B1 [11, 42, 43], the recurrence rate is almost 0 [42, 44]. Adjuvant radiotherapy does not add any advantage for stage II thymoma which is completely resected; and it might be indicated for type B2/B3 lesions [45, 46]. Thus, adjuvant radiotherapy should be reserved in case of close proximity of the tumor to the resection margins or extracapsular involvement of the mediastinal fat or presence of adhesions to the pericardium and mediastinal pleura [8]. Multiple researches show adjuvant radiotherapy makes no benefit for stage III thymoma [11, 42, 47]. Adjuvant radiotherapy should be considered for patients receiving incomplete resection or with suspected involvement of the surgical margins, or with type B2/B3 histology or in case of previous open biopsy that could contribute to contaminate the surgical wound [8]. Besides, some authors suggest a potential role for postoperative chemotherapy, as most recurrence of stage III thymoma are within the pleura [47]. Stage IV patients generally received adjuvant mediastinal radiation therapy, which cannot prevent pleural or pulmonary metastases but may prevent mediastinal recurrence [5]. Surgical removal followed by intrapleural hyperthermic chemotherapy is another treatment strategy [48].

### Follow-up

Patients with incomplete resections and patients with thymic carcinoma are recommended for more frequent (computed tomographic surveillance every 6 months) follow-up during the first 3 years given their higher propensity for relapse [49]. An annual chest CT within 5 years after thymoma resection and biannual chest CTs after 5 years for at least 20 years are recommended [32].

## The treatment of recurrent thymoma

Once the thymoma is relapse, the effect of treatment may not be ideal. As the initial operation usually resect all the thymus and fat tissue in the mediastinum, expose vessels and nerves, so the re-operation is quite risky and difficult. Reducing-tumor operation plus hyperthermia perfusion chemotherapy may be a good choice [50].

Recurrent thymoma is malignant, and multimodal treatment is necessary. Thymoma is a slow glowing tumor, patient may get a long survival without any treatment. Supportive treatment may be the best choice for those who have a low PS score and couldn’t tolerant further treatment.

### Surgery

Most doctors regard surgery as the first choice for patients of recurrent thymoma. The average rates of 5- and 10-year overall survival after recurrence in patients treated surgically were 70.9–82.7 % and 49.6–68.2 %, and in patients treated nonsurgically, they were 29.6–43.5 % and 18.4–25.4 %, respectively [3, 4, 51]. The patients of recurrent thymoma who receive operation have an obvious better overall survival rate than those treated with chemotherapy only [5, 12, 52–55]. Some researchers raised an issue that the operation should be performed as long as the tumor is resectable and the patient could sustain the surgery [29]. Other opinions propose that surgical resection should be considered in patients with a localized recurrence after apparently successful initial therapy; in some patients with stage IV disease, the resection of isolated pleural metastases is an appropriate initial approach; for cases with multiple pleural metastases, chemotherapy, with or without subsequent surgery, is often appropriate [56]. The operative mortality was reported ranging from 0 to 13.3 %, and the operative morbidity ranging from 0 to 32.1 %; most patients suffering from operative mortality and morbidity had myasthenia gravis [57]. So it seems necessary to control myasthenia gravis with drugs pre-operative.

For patients with single pleural recurrences, a partial resection is necessary [16]. Sometimes pleural recurrences are extensive, with huge pleural implants infiltrating the lung and the diaphragm; in such cases, an extended pleuropneumonectomy may be the only chance to attempt radicality [58]. Strict selection criteria for such an aggressive treatment include young age, excellent cardiopulmonary function, and absence of metastatic disease using PET-CT [8]. The type of surgical approach depends upon the site and side of the recurrence, the associated surgical risks for bone infection, and the surgeon’s preference. Pleural recurrences are approached best by means of thoracotomy, although recent reports indicate that sternotomy may be performed; mediastinal recurrence usually is approached by means of median sternotomy. The combination of median sternotomy and anterior thoracotomy may improve exposure of the mediastinum and the ipsilateral hemithorax [8].

Hamaji et al. [17] believe that repeat surgical resection alone has a limited role in the management of recurrent type C thymoma and thymic carcinoid given the higher rate of relapse, earlier relapses, more distant metastases, and lower overall survival and progression-free interval. Other contraindications include: unilateral pleural recurrence with extensive lesions, bilateral pleural recurrence, early recurrence, bilateral pulmonary recurrence, cervical lymph nodes metastasis, extrathoracic recurrence and poor general condition [32].

### Radiotherapy

In nonresectable local recurrences, exclusive radiotherapy has been reported as an efficient treatment, especially on pleural recurrences, even if irradiation had previously been delivered. High response rates with 5-year survival rates as high as 80 % were obtained in small retrospective series. [53].

### Chemotherapy

Many literatures suggest chemotherapy as the best choice for nonresectable recurrent thymoma [52, 59, 60]. However, there isn’t a well-accepted chemotherapy regiment yet. Generally, type C thymoma has the worst sensitivity [61].

Cisplatinum combined with anthracycline is the most popular chemotherapy regiment [62–64]. Taxol is not so effective, but could be the drug of second-line [61]. Besides, cyclophosphamide [63], pemetrexed [65, 66], gemcitabine combined with capecitabine [67] may also help relieve the tumor progression.

An attractive treatment of the pleural implants of thymoma is hyperthermic intrapleural chemotherapy. Circulation of cisplatinum (100 mg/m2) flows of 1000 to 2000 mL/min with an inflow temperature of 42 °C or higher were required to maintain the desired temperature, 79 % of the patients have had complete local control for periods ranging from 10 to 70 months [48]. Cisplatinum combined with adriamycin or doxorubicin are also effective [68, 69].

### Targeted therapy

In recent years, researches on the targeted therapy of recurrent thymoma have started. Epidermal Growth Factor Receptor (EGFR) has a high expression in the cells of thymoma, but mutations are rare, so EGFR tyrosine kinase inhibitors may not be effective. A case report shows that a patient with EGFR mutation fails to respond to gefitinib [70]. However, some EGFR strong positive patients achieve partial remission after treated with cetuximab [71, 72].

Bevacizumab was tested in combination with erlotinib, no tumor response was observed, but stable disease rate was 60 % [73]. 14 % patients could achieve a partial response when treated with cixutumumab, and 76 % have stable disease [74]. 8 % patients achieve partial response under the treatment of histone deacetylase inhibitor Belinostat, and the two-year overall survival rate is 77 % [75].

Somatostatin receptors are expressed in a variety of malignancies including thymic epithelial tumors, so octreotide is regarded as an alternative for recurrent thymoma [76]. In patients whose tumor is octreotide-reactive and treated with octreotide or octreotide combined with prednisone, 28.5–30.3 % show disease response, 35.7–36.8 % have stable disease [77, 78].

### Multimodal treatments

As the initial operation damaged the original anatomical structure, induced local adhesions, and recurrent thymoma is prone to invade main vessels and nerves, so secondary operation is difficult to achieve radical cure, the rate of R0 resection is only 50–60 % [8, 12, 24]. So, post-operative multimodal treatments are necessary for many patients with recurrence. Several researches show that operation followed by other treatments could improve prognosis [17, 49, 79–81]. However, only a small part of patients received treatment after operation (20.4 % for chemotherapy and 20.9 % for radiotherapy) [4]. People have not reach an agreement about the therapeutic regimen yet, and relevant studies are few.

Operation doesn’t achieve R0 resection is regarded as the indication for post-operative adjuvant radiotherapy [17]. The mostly accepted indications to adjuvant radiotherapy are: resection performed macroscopically or microscopically not radical, particularly in case of thymomas, peeled off the phrenic nerve or great vessels; in case of any doubts about radicality of the resection, type B2/3, because it is more aggressive; in case of biopsies through mediastinotomy [16].

Shin et al. [82] revealed an aggressive but highly effective multimodal treatment for locally advanced, unresectable thymoma: induction chemotherapy (three courses of cyclophosphamide, doxolubicin, cisplatin, and prodnisone), surgical resection, postoperative radiation therapy, and consolidation chemotherapy. This multidisciplinary approach may increase the effect of re-operation and elevate their survival rate.

Percutaneous cryoablation is available for patients with pleural recurrence [83]. Other studies show cortisol alone or with tacrolimus is effective for recurrent thymoma [84–86].

### Clinical concerns of recurrent thymoma

The recurrence is of importance to patients of thymoma. The effect of treatment for recurrent thymoma is not as good as primary tumor, and the prognosis is not good. As the recurrent thymoma is relatively rare [2, 11], and disease-free time varies, it is difficult to carry out clinical trial. There are still some clinical concerns about recurrent thymoma.

It’s important and difficult to tell which patients are likely to experience recurrence and prevent the recurrence. Establishing an evaluation system of the recurrent risk is necessary. Patients of high recurrent risk may obtain a benefit through neoadjuvant and adjuvant therapy, including radiotherapy, chemotherapy and targeted therapy. To those who are prone to get recurrent disease, a more frequent than mentioned above [49] maybe meaningful for early intervention.

Many doctors may concern how to achieve the early diagnosis of recurrent thymoma. As there is tissue adhesion, fibrillation and scarring induced by the resection of primary tumor, it may be difficult to diagnose the recurrence by traditional imageological examination. The low recurrent rate and lack of experience is another negative factor. PET-CT shows an advantage in the diagnosis of recurrent thymoma [33, 35]. Its application is limited by the cost, however. Formulating a criteria for the diagnosis is of importance to recurrent thymoma.

Recurrence is an important aspect of thymoma. As there are still concerns about the prevention, diagnosis and management of recurrent thymoma, randomized clinical trials of multi-center is necessary. International collaboration should be encouraged.

## The prognosis of recurrent thymoma

The 5-year and 10-year survival rates are 51–59.9 % and 42.5–43 % [2, 5]; and those of type C thymoma patients are 30.8 and 28.2 % [2]. The 5-year survival rate following complete resection is 64–80 % [43, 44]. Masaoka classification, WHO histology type and complete resection are main factors affecting the prognosis [29, 44, 87]. Early recurrence (<40 months) is known as a negative prognostic factor [15], and local recurrence, single recurrence both imply better prognosis [21, 32, 88]. Regarding the patients with single-site recurrence, the 5- and 10-year survival rates of the patients with pleural dissemination are 90.6 and 66.9 %, which indicate a trend toward a more favorable survival compared with that observed in the patients with recurrence at the other sites [2].

## Conclusion

Recurrent thymoma is rare disease, differences between patients are obvious. It’s difficult to evaluate the regiment of multimodal treatments. Controversy about the management of recurrent thymoma still exists. Multi-center prospective clinical trials with large sample are necessary to provide evidence for making the strategy of managing recurrent thymoma.

## Abbreviations

CT: Computerized tomographic
DNA: Deoxyribonucleic acid
ECT: Emission computed tomography
EGFR: Epidermal Growth Factor Receptor
IFN: Interferon
IL: Interleukin
ITMIG: International Thymic Malignancy Interest Group
JART: Japanese Association for Research on Thymus
MRI: Magnetic resonance imaging
PET-CT: Positron emission tomography
VATS: Video assisted thoracic surgery
WHO: World Health Organization
